# Editorial Policy, DOI and (in)visibility of scientific publications

**DOI:** 10.1590/1518-8345.0000.3732

**Published:** 2022-08-01

**Authors:** Agostinho Antônio Cruz Araújo, Ítalo Rodolfo Silva, Isabel Amélia Costa Mendes

**Affiliations:** 1 Universidade de São Paulo, Escola de Enfermagem de Ribeirão Preto, Centro Colaborador da OPAS/OMS para o Desenvolvimento da Pesquisa em Enfermagem, Ribeirão Preto, SP, Brasil.; 2 Bolsista da Coordenação de Aperfeiçoamento de Pessoal de Nível Superior (CAPES), Brasil.; 3 Universidade Federal do Rio de Janeiro, Campus Macaé, Macaé, RJ, Brasil.

Rescuing and valuing already produced science is fundamental. From this understanding, we can infer the surveillance degree necessary for strengthening network science, which must be visible, easily accessible and perennial in its web formats, in addition to presenting favorable conditions for the scientometric, authenticity and ethical parameters in the productions published and widely disseminated in the virtual environment.

Such being the case, publishing, communicating and publicizing scientific knowledge are stages that support not only the researchers’ responsibility, but also the journals’ in their commitment to the current scientific community, as well as to future researchers.

From this perspective, the scientific publishing area follows and responds to the speed of technological innovations and social transformations. Thus, we saw the emergence of a new publishing venture in the digital environment, whether in free or commercial modalities, which allows interested readers to have immediate, full, available and publicized access, in increasingly innovative and changeable formats, to the extent that social communication evolves and editors of scientific journals assume and implement changes in the way of placing their products within reach of potential interested parties.

Among the needs arising from the migration of scientific publications from print to digital in most scientific journals is the one focusing on the importance of keeping productions locatable and secure, with development of strategies that have been implemented as a way of identifying scientific research studies as a result of this requirement, such as the Digital Object Identifier (DOI). This service is adopted at the global level and provides information standardization and preservation of the published material^1^ ^-^ ^2^.

Creation of the DOI in the late 1990s emerged from discussions in the publishing industry that recognized the need to uniquely identify published contents^2^ ^-^ ^3^. In this movement, the first record made through this unique identifier occurred in 2000, by the Crossref Registration Agency^2^. Since then, the number of records has grown significantly and, currently, this practice is widespread and used worldwide.

Consisting of numbers and letters, DOIs therefore help locate scientific productions. Thus, the main formatting styles for references, such as Vancouver, the American Psychological Association (APA) Style and the American Medical Association (AMA) Manual Style assign a DOI attached to the reference, complementing it, in order to favor location and immediate access to the cited production. The result of this process is not only identification but also reliability for accessing diverse information widely disseminated in the digital environment.

In addition, identifiers have some related concepts which highlight their importance, namely: uniqueness, resolution, interoperability and persistence^3^, where the last characteristic is emphasized, considering that, from the moment an identifier is assigned, there will always be a reference to the cited material and, in this way, representing a connection with the future.

When considering scientific progress, the importance of previous knowledge produced in science is emphasized and, therefore, in the conceptualization of new research studies. In this sense, although the first record through a DOI only took place in 2000^2^ ^-^ ^3^, it is noted that there is the possibility of registering previous articles, called “back file”.

In the scientific community, there is consensus about the understanding on where the importance of current research studies for the development of science lies. Such concern is pertinent to social dynamics, which is equally transitory and complex. However, prioritizing the records of productions prior to the period in which the journals adopted DOI use signals a commitment to the process for developing science today, aiming at valuing the science of the future: robust, perennial and with identity forged in its own historicity.

In fact, this discussion is fundamental, as DOIs can be one of the ways to consolidate a journal in databases indexes to obtain better bibliometric indices and, therefore, expand its visibility in front of other scientific journals. This is a stance that should be reflected by the Nursing editorial community, in the reiteration of its commitment to Nursing Science itself.

In addition, considering that this strategy is used at the global level, it becomes necessary to ask the following question: Is there any gap in its use? The answer is related to the commitment to scientific publishing inherent to each journal; although some of them adopt the digital identifier as a policy for comprehensive collection, it is noticed that, in most of the cases, this strategy is still restricted to recently published articles. Thus, it is also imperative to value previous scientific literature: the basis of current knowledge.

Consequently, we defend the need to adopt an equality principle in rescuing and valuing science, reason why we recommend retroactive implementation of digital identification through DOIs. This measure ensures not only an accelerated dynamics of the parameters for good editorial practices but also the configuration of journals as guardians of scientific knowledge, which must be perennial in its path so that we can achieve a real understanding of the current and future challenges in Nursing Science. 

## References

[1] Gorraiz J, Melero-Fuentes D, Gumpenberger C, Valderrama-Zurián JC (2016). Availability of digital object identifiers (DOIs) in Web of Science and Scopus. J Informetr.

[2] International DOI Foundation (2015). DOI® Handbook.

[3] Paskin N, Bates MJ, Maack MN (2010). Encyclopedia of Library and Information Sciences.

